# Assessing the prognostic value of stemness-related genes in breast cancer patients

**DOI:** 10.1038/s41598-020-73164-3

**Published:** 2020-10-27

**Authors:** Wen-Jie Wang, Han Wang, Meng-sen Wang, Yue-Qing Huang, Yu-Yuan Ma, Jie Qi, Jian-Ping Shi, Wei Li

**Affiliations:** 1 Department of Radio-Oncology, The Affiliated Suzhou Hospital of Nanjing Medical University, 26 Daoqian Street, Suzhou, 215001 Jiangsu People’s Republic of China; 2 Department of Oncology, Jining Tumor Hospital, Jining, 272000 Shandong People’s Republic of China; 3 Department of Oncology, Jining No. 1 People’s Hospital, Jining, 272011 Shandong People’s Republic of China; 4 Department of General Practice, The Affiliated Suzhou Hospital of Nanjing Medical University, Suzhou, 215001 People’s Republic of China; 5 Department of Thyroid and Breast Surgery, The Affiliated Suzhou Hospital of Nanjing Medical University, Suzhou, 215001 Jiangsu People’s Republic of China; 6 Department of Oncology, The First Affiliated Hospital of Soochow University, 188 Shizi Street, Suzhou, 215006 Jiangsu People’s Republic of China

**Keywords:** Cancer, Computational biology and bioinformatics, Stem cells, Oncology

## Abstract

Breast cancer (BC) is currently one of the deadliest tumors worldwide. Cancer stem cells (CSCs) are a small group of tumor cells with self-renewal and differentiation abilities and high treatment resistance. One of the reasons for treatment failures is the inability to completely eliminate tumor stem cells. By using the *edgeR* package, we identified stemness-related differentially expressed genes in GSE69280. Via Lasso-penalized Cox regression analysis and univariate Cox regression analysis, survival genes were screened out to construct a prognostic model. Via nomograms and ROC curves, we verified the accuracy of the prognostic model. We selected 4 genes (*PSMB9*, *CXCL13*, *NPR3*, and *CDKN2C*) to establish a prognostic model from TCGA data and a validation model from GSE24450 data. We found that the low-risk score group had better OS than the high-risk score group, whether using TCGA or GSE24450 data. A prognostic model including four stemness-related genes was constructed in our study to determine targets of breast cancer stem cells (BCSCs) and improve the treatment effect.

## Introduction

Breast cancer (BC) is one of the most common tumors in females. In China, the number of new cases is 272,400, and the death toll is 69,500 every year^1^. According to immunohistochemical analysis, BC can be divided into luminal-type, Her2-positive and triple-negative breast cancer (TNBC), of which TNBC has the worst prognosis^2,3^. With improved treatment, the mortality rate of BC is decreasing year by year^4,5^, but 70% of patients have recurrence and metastasis within 5 years^6^.

There is a small group of stem-like cells in tumors called cancer stem cells (CSCs). CSCs have the characteristics of self-renewal and differentiation abilities and high drug resistance^7–11^. Previous studies have indicated that this portion of breast cancer stem cells (BCSCs) is identified by cell surface markers, such as *CD44*, *CD24*, *CD133* and *ALDH*^12,13^. With changes in the tumor microenvironment, BC cells can differentiate into tumor stem-like cells^14,15^. In BC-resistant cell lines and tissues, the CSC population is significantly increased by chemotherapy^16^. Compared with other types of BC, TNBC has the highest expression of stem cell markers, which may be one of the reasons for TNBC having the worst prognosis^15,17^. Previous studies have shown that stemness-related-gene expression can be used as a predictive biomarker for breast cancer patients. Akbar et al. identified a novel gene list (CNCL) that can discern the stemness and EMT phenotypic statuses of breast cancer, thereby tracking tumor cells and altering the response to tumor treatment^18^.

Treatment for BCSCs has already emerged but is still immature. In our article, we hope to identify multiple stemness-related genes for determining BC prognosis by establishing a prognostic model. These genes may be potential targets for treating breast cancer, which may improve patient survival.

## Result

### Selected stemness-related differentially expressed genes (DEGs)

Via *edgeR,* we identified 599 stemness-related DEGs in GSE69280; among them, 255 genes were upregulated, and 344 genes were downregulated, with thresholds of |log_2_ FC|> 1.0 and *P* < 0.05 (Fig .S2).

### Identification of stemness-related DEGs in the TCGA BC database

**Figure 1:**
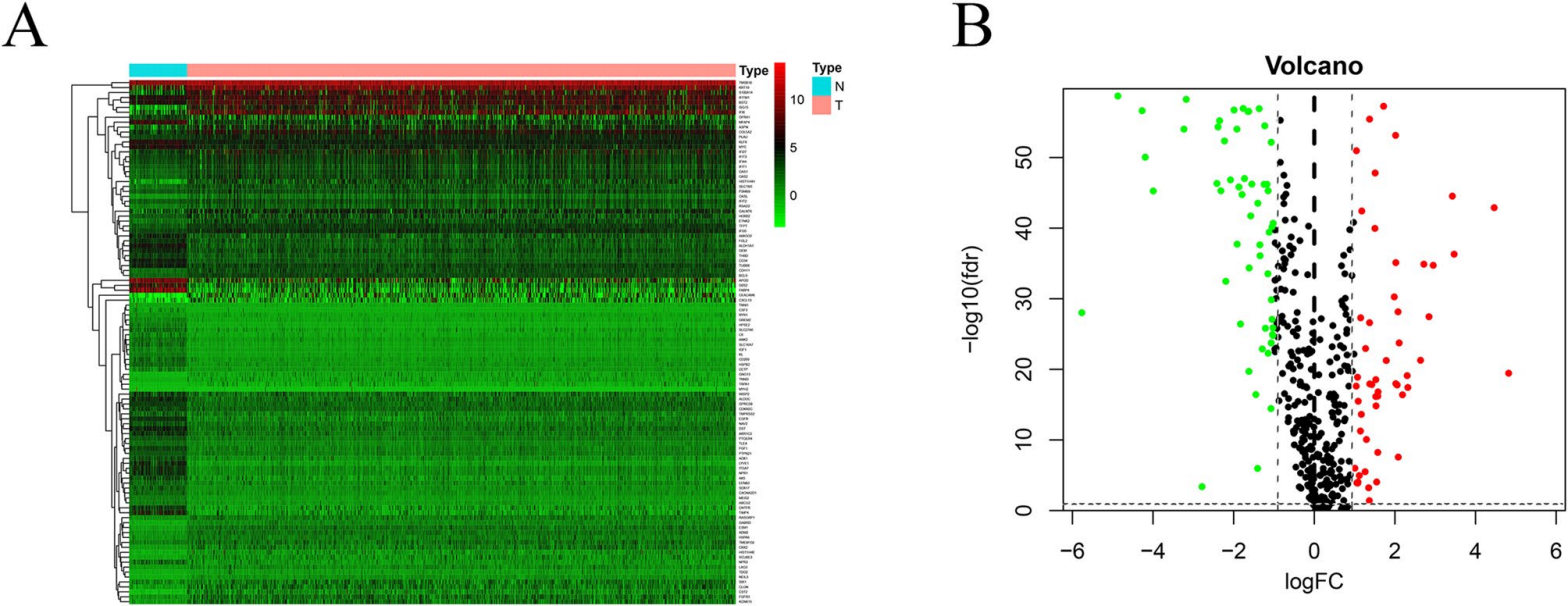
The stemness-related differentially expressed genes of breast cancer patients. (**A**) Heatmap and (**B**) Volcano plot.

Coexpressed genes were obtained by intersection of TCGA and GSE24450 data. We obtained 566 genes by intersection the stemness-related DEGs list and TCGA data. By Using *edgeR*, we identified 106 stemness-related DEGs in TCGA BC patients; among them, 54 genes were downregulated, and 52 genes were upregulated, with thresholds of |log_2_ FC|> 1.0 and an adjusted *P* < 0.05 (Fig. 1A,B).

### Construction of the stemness-related-gene prognostic model

**Figure 2:**
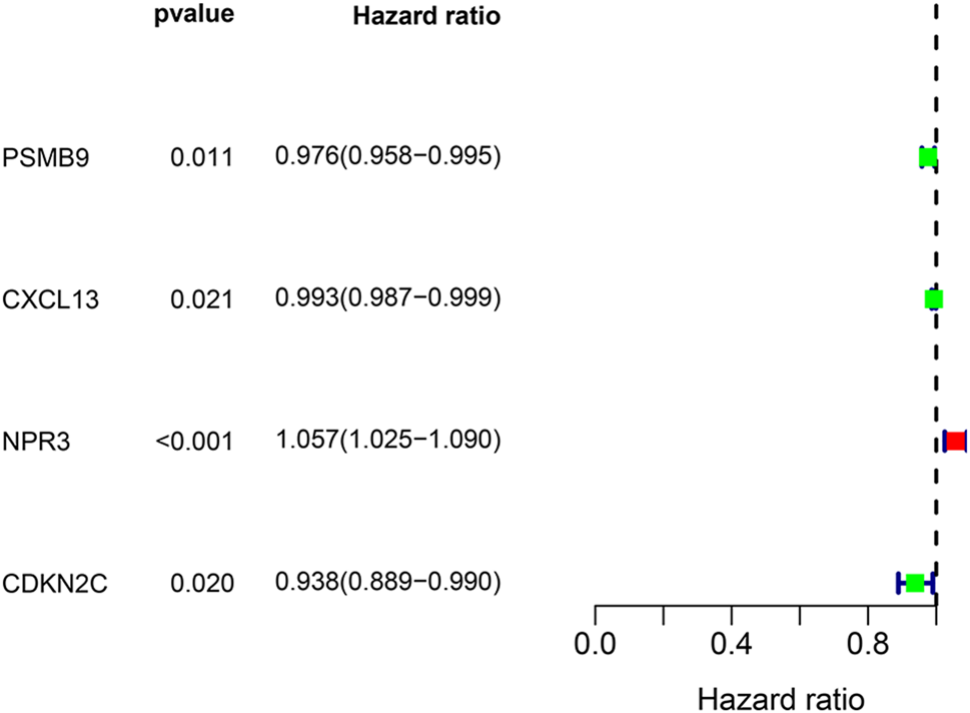
The survival-associated stemness-related differentially expressed genes of breast cancer patients.

**Figure 3:**
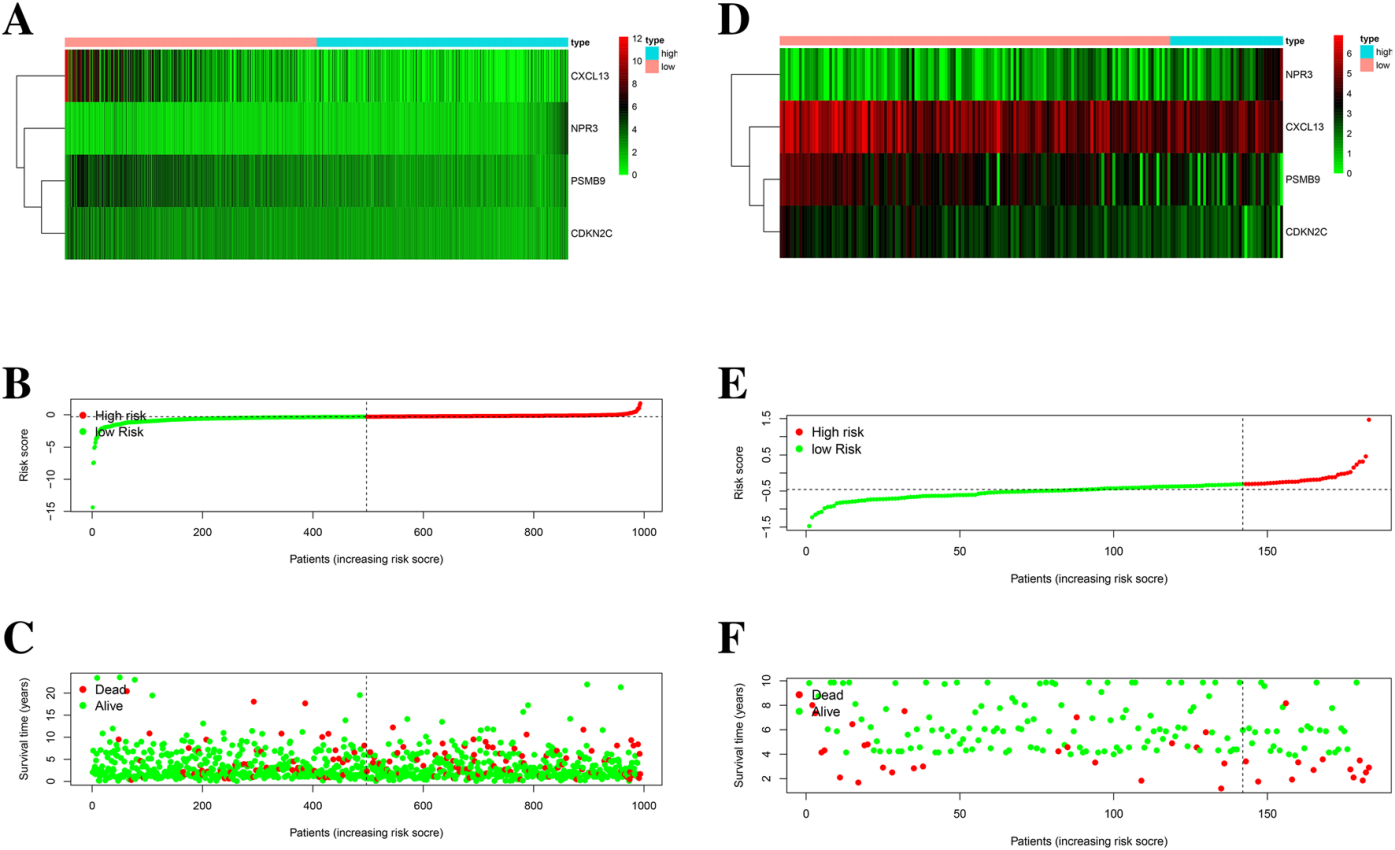
Establishment of the stemness-related prognostic model. (**A**) Heatmap of four genes in the TCGA model. (**B**) Rank of risk score and distribution of groups in the TCGA data. (**C**) Survival status of TCGA BC patients in different groups. (**D**) Heatmap of four genes in the GSE24450 model. (**E**) Rank of risk score and distribution of groups in the GSE24450 data. (**F**) Survival status of GSE24450 BC patients in different groups.

By using univariate Cox regression analysis, we obtained the survival-associated genes shown in Fig. 2. Lasso-penalized Cox regression was performed to identify the genes in the prognostic model. We constructed a prognostic model and used GSE24450 to build a validation model. In TCGA prognostic model the expression of genes for each patient is shown in Fig. 3A, the distribution of different risk scores is shown in Fig. 3B, the distribution of different survival statuses (years) of TCGA patients is shown in Fig. 3C. In GSE24450 validation model the expression of genes for each patient is shown in Fig. 3D, the distribution of different risk scores is shown in Fig. 3E, the distribution of different survival statuses (years) of GSE24450 patients is shown in Fig. 3F. The risk score for the prognostic gene signature was calculated as follows: risk score = (expression level of *PSMB9* × − 0.01623) + (expression level of *CXCL13* × − 0.00335) + (expression level of *NPR3* × 0.05481) + (expression level of *CDKN2C* × − 0.04691).

**Figure 4:**
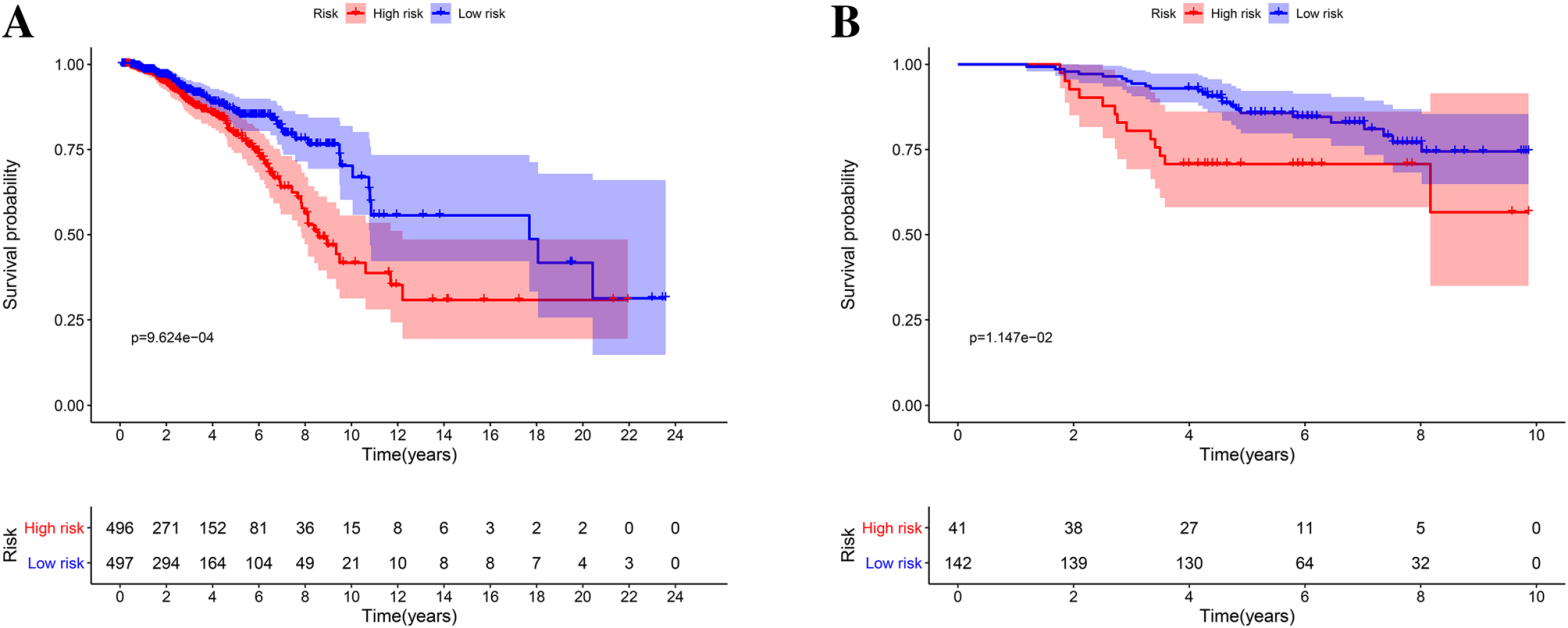
Survival analysis of the prognostic models. (**A**) The KM curve of the TCGA model. (**B**) The KM curve of the GSE24450 model.

We classified patients into low- and high-risk score groups based on the median risk score as the cut-off. Survival was analyzed by a Kaplan–Meier (KM) curve, and the low-risk-score group had better overall survival (OS) than the high-risk-score group (*P* < 0.001) (Fig. 4A). In the validation model, the low-risk-score group had better OS than the high-risk-score group (*P* = 0.0115) (Fig. 4B).

### The clinical utility of the prognostic model

**Figure 5:**
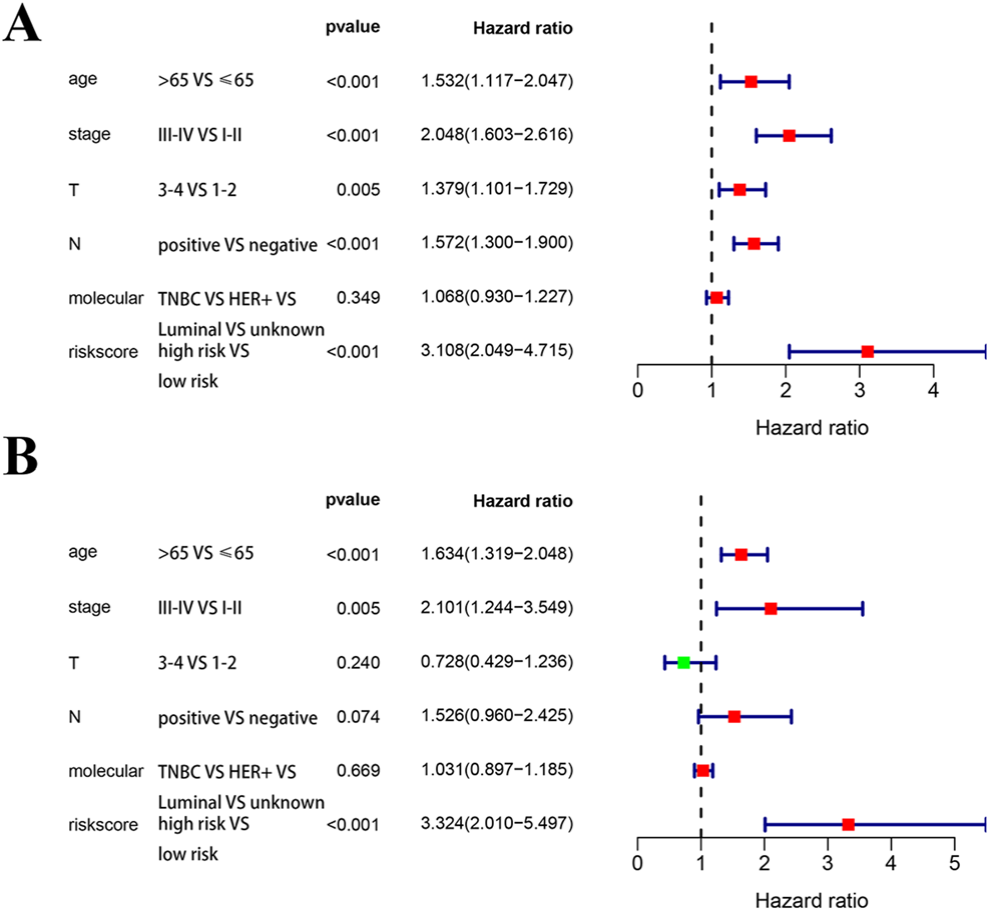
Cox regression analyses of the prognostic model and clinical features. (**A**) Univariate Cox analyses of the TCGA model. (**B**) Multivariate Cox regression analysis of the TCGA model.

**Figure 6:**
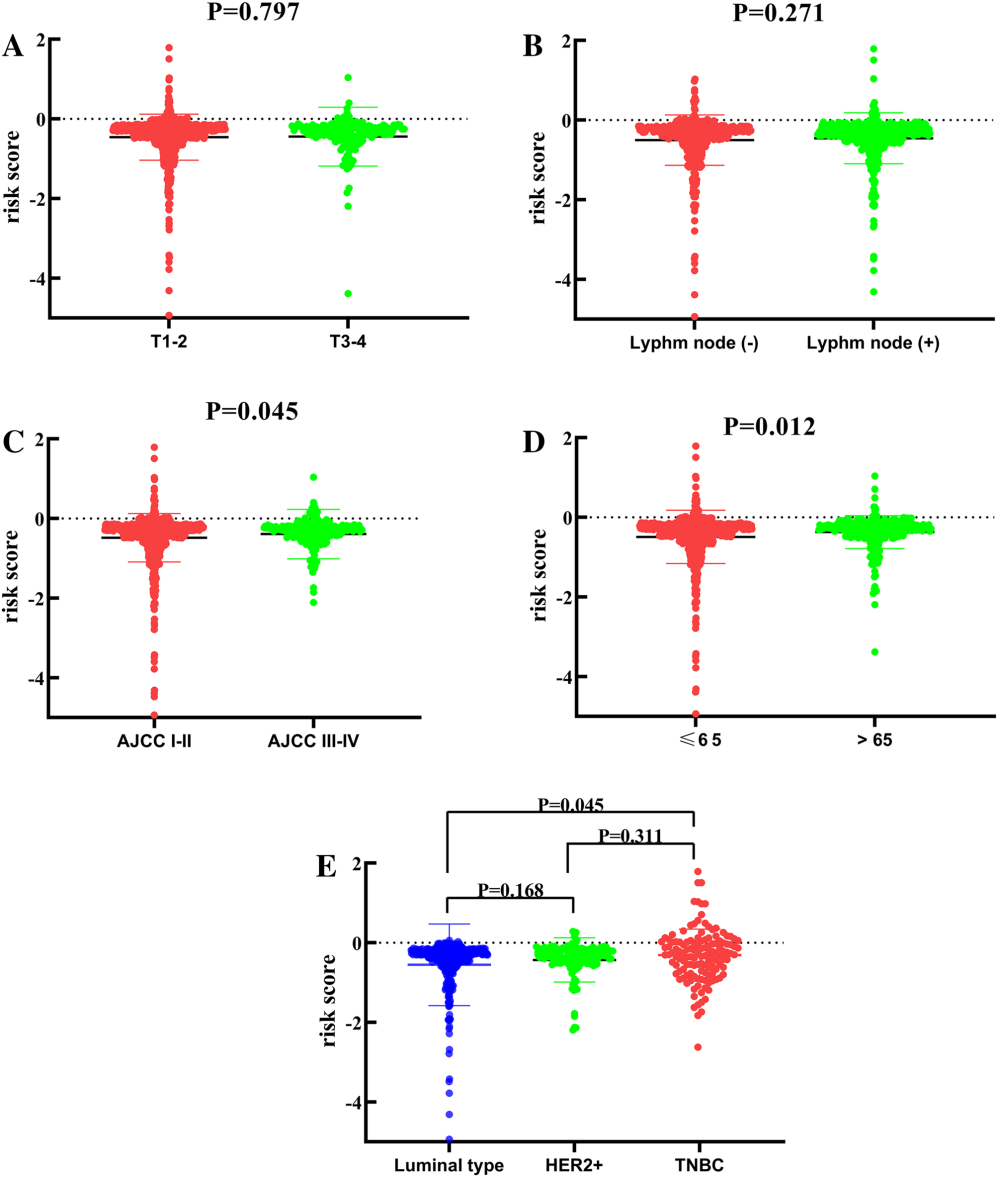
The relationship between risk score and clinical features. (**A**) The risk score in different T stage groups. (**B**) The risk score in different lymph node metastasis groups. (**C**) The risk score in different AJCC stage groups. (**D**) The risk score in different age groups. (**E**) The risk score in different molecular phenotype groups.

In the TCGA prognostic model, univariate Cox regression analyses (Fig. 5A) showed that older age (> 65) (hazard ratio [HR 1.532; 95% confidence interval [CI] = 1.117–2.047; *P* < 0.001), high American Joint Committee on Cancer (AJCC) stage (III-IV) (HR = 2.048; 95% CI = 1.603–2.616; *P* < 0.001), high tumor (T) stage (3–4) (HR = 1.379; 95% CI = 1.101–1.729; *P* = 0.005), lymph node metastasis (positive) (HR = 1.572; 95% CI = 1.300–1.900; *P* < 0.001), and high risk score (HR = 3.108; 95% CI = 2.049–4.715; *P* < 0.001) were significant risk factors for poor prognosis. In the multivariate Cox regression analysis (Fig. 5B), older age (> 65) (HR = 1.634; 95% CI = 1.319–2.048; *P* < 0.001), high AJCC stage (III–IV) (HR = 2.101; 95% CI = 1.244–3.549; *P* = 0.005) and high risk score (HR = 3.324; 95% CI = 2.010–5.497; *P* < 0.001) were found to be independently associated with poor OS. The risk scores were significantly higher for patients with higher AJCC stage (III-IV) (Fig. 6C) and older age (> 65) (Fig. 6D). The risk score was significantly higher in TNBC patients than in luminal-type patients (Fig. 6E). The risk scores for different T stages (Fig. 6A) and different lymph node statuses (Fig. 6B) were not statistically significantly different. The risk scores in luminal-type patients and HER2-positive patients were not statistically significantly different (Fig. 6E). The risk scores in HER2-positive patients and TNBC patients were not statistically significantly different (Fig. 6E).

### Verification of the accuracy of the prognostic model

**Figure 7:**
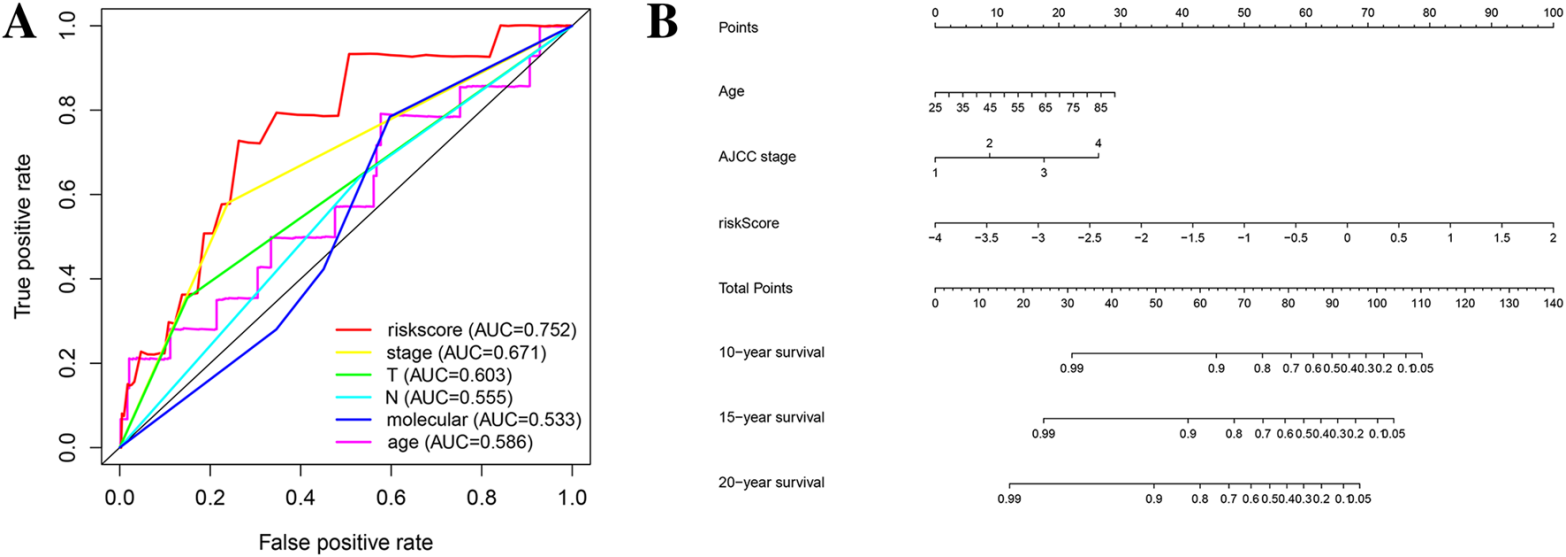
Verification of the accuracy of prognostic models. (**A**) The ROC curve of the TCGA prognostic model. (**B**) The nomogram of the TCGA prognostic model.

To further verify the accuracy of the prognostic model, we constructed a nomogram and ROC curve. The ROC curve analysis of the TCGA prognostic model is shown in Fig. 7A, and the area under the curve (AUC) was 0.752. The nomogram is shown in Fig. 7B, and the C-index was 0.758.

### Functional enrichment analysis of stemness-related genes

**Figure 8:**
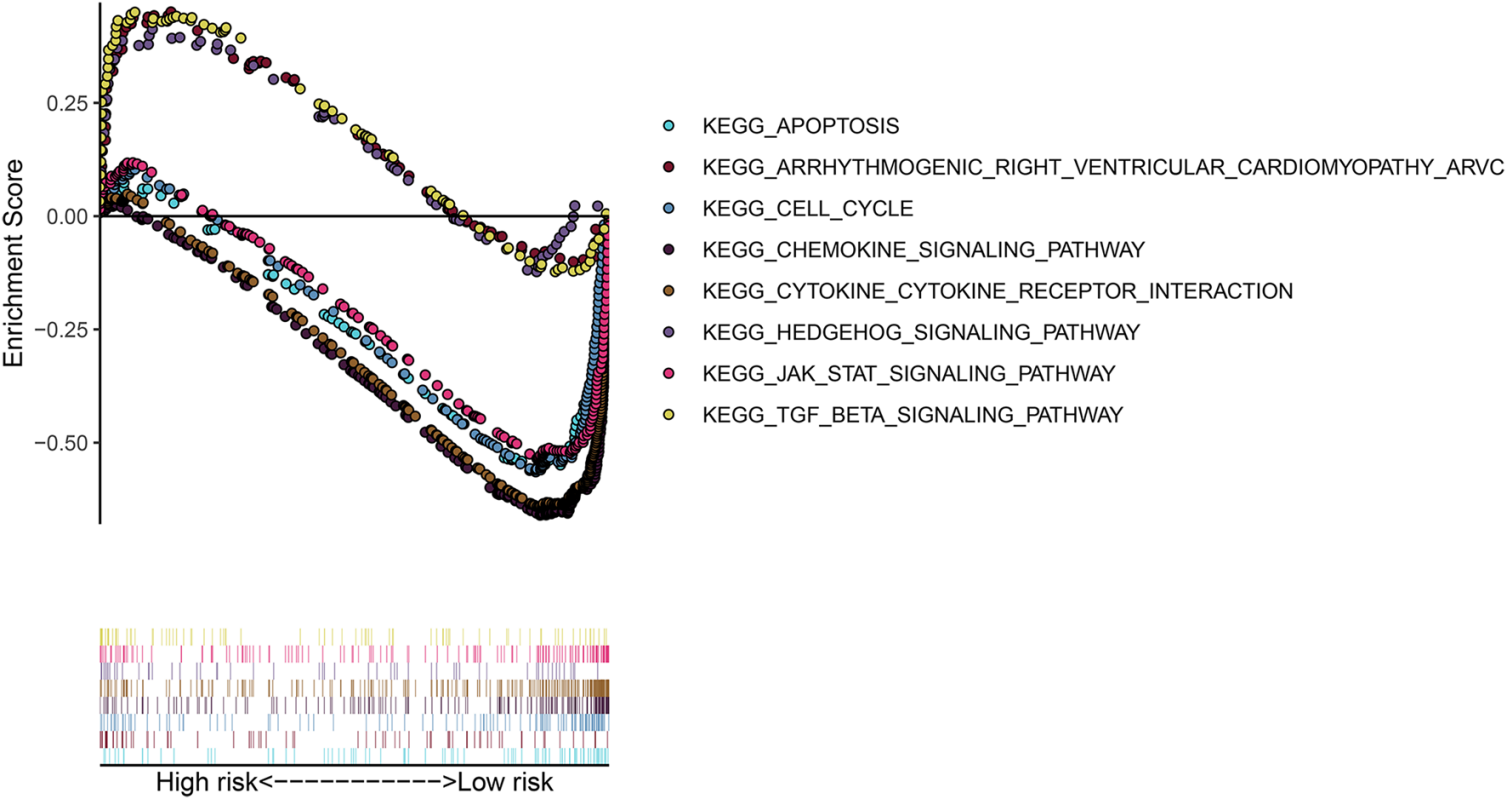
KEGG pathway enrichment analysis.

Through GSEA, we found that the high-risk-score group had enrichment in KEGG pathways related to metabolism (Fig. 8): the hedgehog signaling pathway, the TGF-β signaling pathway and a pathway related to arrhythmogenic right ventricular cardiomyopathy (ARVC). The low-risk-score group had enrichment in the following KEGG pathways (Fig. 8): the cell cycle, apoptosis, chemokine, cytokine and JAK-STAT pathways.

## Discussion

In this research, we identified DEGs with potential stemness characteristics by analyzing stem-like and non-stem-like cells in GSE69280. Then, the DEGs were compared with TCGA and GSE24450 data to select coexpressed genes in the two databases. Next, by using univariate Cox regression analysis and Lasso-penalized Cox regression analysis, we obtained four prognostic-related genes (*PSMB9*, *CXCL13*, *NPR3*, and *CDKN2C*) and established a prognostic model. The model was validated with GSE24450 data. We divided patients into low-risk-score and high-risk-score groups and found that the low-risk-score group had better OS than the high-risk-score group for both TCGA and GSE24450 data.

BCSCs are a small group of tumor cells that have self-renewal capacity and play an important role in tumor formation, recurrence and metastasis^19^. Furthermore, resistance to traditional chemoradiotherapy is a remarkable feature of BCSCs, as well as one of the culprits for treatment failure^20^. Recent studies have demonstrated that breast non-stem cells undergo dedifferentiation and transform into CSCs in response to treatment^21^. In addition, traditional treatments cannot thoroughly eliminate BCSCs, which contributes to a significant increase in the proportion of CSCs^22^. The main reasons for the resistance of CSCs are as follows. First, CSCs inhibit the expression of membrane-bound APC transporters, which act as efflux drug pumps to decrease intracellular drug accumulation^20^. In addition, CSCs also have DNA repair and antiapoptotic effects^23^, which are responsible for resistance to treatment. What’s more, different BC molecular subtypes, such as TNBC cells and HER-2 positive cells, has the similar stemness, but they are tow unique diseases that require different treatment strategies^24^. In BCSCs of different molecular subtypes, the expression and regulation of HER-2 are both different, so therapeutic repercussion and prognosis of patients will be different^25^. Thus, elimination of BSCSs is a potential new strategy for patients with refractory breast cancer.

Our prognostic model was constructed with a series of survival-associated DEGs, including *PSMB9*, *CXCL13*, *NPR3*, and *CDKN2C*. *CDKN2C*, also known as *p18* or *INK4C*, is a member of the INKCK family and regulates the G1 phase of the cell cycle by inhibiting *CDK4* or *CDK6*^26^. Previous studies have reported that *CDKN2C* is involved in the regulation of normal stem cells and CSCs^27^. Yuan et al*.* pointed out that liver CSC counts significantly increased in the absence of *CDKN2C* expression, suggesting that *CDKN2C* strongly inhibited the self-renewal of liver CSCs^28^. Gain of the *CCND1* and *CDK4* and loss of the *CDKN2A* (*p16*) and *CDKN2C* (*p18*) genes are present in patients with luminal B breast cancer and poor prognosis of and negatively regulated by the cell cycle pathway^29^. Currently, inhibitors targeting *CDK4/6* have been clinically approved for breast cancer patients who have failed hormone receptor-targeted treatment. *CXCL13* is a member of the chemokine family and is an important component of the tumor microenvironment. In vivo, *IL-30* overexpression in primary tumors facilitates the recruitment of prostate cancer stem-like cells (PCSLCs) to *CXCL13*, creating a microenvironment convenient for lymph node and blood metastasis^30,31^. Zhang found that mesenchymal stem cells (MSCs) could secrete a large amount of *CXCL13* in the bone marrow microenvironment of multiple myeloma and promote the proliferation, metastasis and drug resistance of myeloma cells through a *CXCL13*-mediated signaling pathway^32^. *PSMB9* is one of the genes encoding proteasome subunits in human embryonic stem cells (hESCs) and plays a key role in maintaining the pluripotency of hESCs and regulating the cell cycle^33^. *NPR3* is enriched in bone marrow mesenchymal stem cells (BM-MSCs) and has important regulatory effects on BM-MSCs^34^. Therefore, considering the regulatory role of these four stemness-related genes, our prognostic signature might be a potential biomarker in breast cancer outcome prediction.

GSEA revealed that the high-risk-score group was enriched in the Hedgehog, TGF-β and cardiovascular KEGG pathways, while the low-risk-score group was enriched in the cell cycle, apoptosis, chemokine, cytokine and JAK-STAT KEGG pathways. The Hedgehog signaling pathway is essential for maintenance of BCSCs^35^, and inhibition of the components of the Hedgehog signaling pathway, such as Gli1, Gil2 and SHH, can reduce CSCs in breast cancer cell lines^35,36^. The components of the tumor microenvironment (cytokines, chemokines, and exosomes)^37,38^ and multiple signaling pathways, such as the apoptotic pathway^39^ and the cell cycle pathway^27^, both play an important role in maintaining the phenotype and function of CSCs. The JAK2-STAT pathway mediates BCSC resistance^40^, while JAK1-STAT may participate in non-CSC transformation into BCSCs^41^. Thus, the KEGG pathways involved in both groups are closely related to maintaining stemness, which may provide strategies for BC treatment. There are already some clinical trials that act directly on the hedgehog, Notch and Wnt signaling pathways and have some effects on CSC suppression^42–44^. However, unfortunately, although there are treatment strategies for CSCs, the translational of these treatments into the clinic for BC patients has been unsatisfactory.

There are some limitations in our present study. For example, the selected genes have been demonstrated to play an important role in maintaining CSCs or other SCs (BM-MSCs and hESCs), some of which have different roles in breast cancer, but few studies have involved the relationship of these genes with BCSCs. This requires further research in the future.

A prognostic model consisting of stem cell-associated genes was constructed in our study. In data from both TCGA and GSE24450, the low-risk-score group had worse outcomes than the high-risk-score group. Although BCSCs account for only a small proportion of all breast cancer cells, these cells play an important role in the recurrence and metastasis of the disease, and traditional treatment cannot thoroughly eliminate them. To the best of our knowledge, this is the first study to build a stemness-related prognostic signature in BC. It is hoped that our present study can provide potential biomarkers for BC outcome prediction and targets for therapies.

## Material and methods

### Selected stemness-related DEGs

Via the *edgeR* package (v3.53) (https://bioconductor.org/packages/edgeR/) (R Development Core Team, Vienna, Austria), we analyzed the GSE69280 data in cells with stemness characteristics and cells without stemness characteristics and identified the stemness-related DEGs (with thresholds of |log_2_ fold change [FC]|> 1.0 and false discovery rate [FDR] adjusted to *P* < 0.05).

### Data collection

Patient clinical information and mRNA sequencing data were obtained from The Cancer Genome Atlas (TCGA) and GSE24450. The TCGA database contains 1066 BC tissues and 112 adjacent normal tissues, the clinical features of patients were showed in Table 1. GSE24450 included 183 breast cancer patients. All patients had complete survival information, and the follow-up time was more than 10 years; the validation data set had similar characteristics. The DEGs were identified as follows: (A) First, the coexpressed genes were obtained by intersecting TCGA and GSE24450 genes. (B) Second, a stemness-related gene list was obtained from GSE69280. (C) Next, the DEGs in BC samples from TCGA were identified. (D) Finally, we compared the stemness-related gene list and TCGA DEGs to obtain eligible stemness-related DEGs. The flow chart is shown in Fig S1.

**Table 1:** The clinical features of TCGA breast cancer patients.

Clinical features		
Age (years)	Media	58
	Rage	26–89
	Numbers of patients (n = 1066)	Numbers of patients (%)
Gender	Female	1055 (98.97)
	Male	11 (1.03)
T stage	T1	280 (26.27)
	T2	617 (57.88)
	T3	131 (12.29)
	T4	36 (3.38)
	Unknown	2 (0.18)
N stage	0	501 (47.00)
	1	358 (33.58)
	2	118 (11.69)
	3	74 (6.94)
	Unknown	15 (0.79)
M stage	0	888 (83.80)
	1	19 (1.78)
	Unknown	159 (14.42)
AJCC stage	I	183 (17.17)
	II	602 (56.47)
	III	240 (22.51)
	IV	19 (1.78)
	Unknown	20 (2.07)
HER-2 status	Positive	156 (14.63)
	Negative	560 (52.53)
	Unknown	350 (32.84)
Estrogen receptor status	Positive	787 (73.83)
	Negative	233 (21.86)
	Unknown	46 (4.31)

AJCC, American Joint Committee on Cancer; HER-2, human epidermal growth factor receptor-2; M, metastasis, N, node; T, tumor; TCGA, The Cancer Genome Atlas.

### Identification of stemness-related differentially expressed genes (DEGs)

Through the *R* limma package^45^, we identified stemness-related DEGs for BC in the TCGA data (with thresholds of |log_2_ fold change (FC)|> 1.0 and false discovery rate [FDR] adjusted to *P* < 0.05).

### Establishment of a prognostic model and validation model

Prognostic risk scores were obtained for all patients by univariate Cox regression analysis and Lasso-penalized Cox regression^46^. The risk score calculation formula for all patients is as follows.

$$ Survival\;Risk\;Score\, \left( {SRS} \right) = \mathop \sum \limits_{i = 1}^{n} \left( {C_{i} \times V_{i} } \right) $$

In the formula, *n* represents the number of mRNAs, *C*_*i*_ represents the coefficient of the mRNA in multivariate Cox regression analysis, and *V*_*i*_ represents the expression level of the mRNA.

Patients were classified into a high-risk-score group and a low-risk-score group by median risk score. To further verify the feasibility of the prognostic model, we also divided GSE24450 patients into two groups according to the median risk score. The survival of the two groups of patients was analyzed by KM curves.

### Construction of a prognosis-related nomogram and receiver operating characteristic (ROC) curves

To further verify the accuracy of the prognostic model, a nomogram and ROC curves were established by the edgeR package^47,48^. The C-index was used to evaluate the accuracy of the nomogram by a bootstrap method with 1000 resamples.

### Functional enrichment analysis

To better understand the underlying biological mechanisms of these genes, KEGG pathway analyses were performed (gene set enrichment analysis [GSEA])^49^. KEGG pathway analyses were based on a threshold of *P* < 0.05.

### Statistical analysis

Statistical analyses were performed by using GraphPad Prism (version 8.0, San Diego, USA). Independent prognostic factors were determined by using a multivariate Cox regression model. Patient survival time was analyzed using the KM curve, and the log-rank test was used for statistical analysis. *P* < 0.05 was considered to indicate a statistically significant difference.

### Ethics declarations

Our research is in compliance with the Declaration of Helsinki.

## Supplementary information

Supplementary Figures.

## Data Availability

The datasets generated and analyzed during the current study are available in the TCGA and GEO repositories.

## References

[1] Chen W (2016). Cancer statistics in China, 2015. CA Cancer J. Clin..

[2] Prat A, Perou CM (2011). Deconstructing the molecular portraits of breast cancer. Mol. Oncol..

[3] Ovcaricek T, Frkovic SG, Matos E, Mozina B, Borstnar S (2011). Triple negative breast cancer—prognostic factors and survival. Radiol. Oncol..

[4] Njor S (2012). Breast cancer mortality in mammographic screening in Europe: a review of incidence-based mortality studies. J. Med. Screen..

[5] Toriola AT, Colditz GA (2013). Trends in breast cancer incidence and mortality in the United States: implications for prevention. Breast Cancer Res. Treat..

[6] Gupta PB (2009). Identification of selective inhibitors of cancer stem cells by high-throughput screening. Cell.

[7] Singh SK (2004). Identification of human brain tumour initiating cells. Nature.

[8] Kim CF (2005). Identification of bronchioalveolar stem cells in normal lung and lung cancer. Cell.

[9] Chen K, Huang YH, Chen JL (2013). Understanding and targeting cancer stem cells: therapeutic implications and challenges. Acta Pharmacol. Sin..

[10] Peitzsch C, Tyutyunnykova A, Pantel K, Dubrovska A (2017). Cancer stem cells: the root of tumor recurrence and metastases. Semin. Cancer Biol..

[11] Baccelli I (2013). Identification of a population of blood circulating tumor cells from breast cancer patients that initiates metastasis in a xenograft assay. Nat. Biotechnol..

[12] Ricardo S (2011). Breast cancer stem cell markers CD44, CD24 and ALDH1: expression distribution within intrinsic molecular subtype. J. Clin. Pathol..

[13] Gourlay SG, McNeil JJ (1990). Antismoking products. Med. J. Aust..

[14] Chaffer CL (2013). Poised chromatin at the ZEB1 promoter enables breast cancer cell plasticity and enhances tumorigenicity. Cell.

[15] Koren S (2015). PIK3CA(H1047R) induces multipotency and multi-lineage mammary tumours. Nature.

[16] Phillips TM, McBride WH, Pajonk F (2006). The response of CD24(-/low)/CD44+ breast cancer-initiating cells to radiation. J. Natl. Cancer Inst..

[17] Liu R (2007). The prognostic role of a gene signature from tumorigenic breast-cancer cells. N. Engl. J. Med..

[18] Akbar MW (2020). A stemness and EMT based gene expression signature identifies phenotypic plasticity and is a predictive but not prognostic biomarker for breast cancer. J. Cancer.

[19] Al-Hajj M, Wicha MS, Benito-Hernandez A, Morrison SJ, Clarke MF (2003). Prospective identification of tumorigenic breast cancer cells. Proc. Natl. Acad. Sci. USA.

[20] Pavlopoulou A (2016). Determinants of resistance to chemotherapy and ionizing radiation in breast cancer stem cells. Cancer Lett..

[21] Lagadec C, Vlashi E, Della Donna L, Dekmezian C, Pajonk F (2012). Radiation-induced reprogramming of breast cancer cells. Stem Cells.

[22] Wang Y (2014). Blocking the formation of radiation-induced breast cancer stem cells. Oncotarget.

[23] Sotiropoulou PA, Christodoulou MS, Silvani A, Herold-Mende C, Passarella D (2014). Chemical approaches to targeting drug resistance in cancer stem cells. Drug Discov. Today.

[24] Mei Y, Cai D, Dai X (2020). Modulating cancer stemness provides luminal a breast cancer cells with HER2 positive-like features. J. Cancer.

[25] Voutsadakis IA (2019). HER2 in stemness and epithelial-mesenchymal plasticity of breast cancer. Clin. Transl. Oncol..

[26] Sherr CJ, Roberts JM (1995). Inhibitors of mammalian G1 cyclin-dependent kinases. Genes Dev..

[27] Cheng T (2004). Cell cycle inhibitors in normal and tumor stem cells. Oncogene.

[28] Yuan Y, Shen H, Franklin DS, Scadden DT, Cheng T (2004). In vivo self-renewing divisions of haematopoietic stem cells are increased in the absence of the early G1-phase inhibitor, p18INK4C. Nat. Cell Biol..

[29] Cancer Genome Atlas Network (2012). Comprehensive molecular portraits of human breast tumours. Nature.

[30] Meijer J, Zeelenberg IS, Sipos B, Roos E (2006). The CXCR5 chemokine receptor is expressed by carcinoma cells and promotes growth of colon carcinoma in the liver. Cancer Res..

[31] Massague J, Obenauf AC (2016). Metastatic colonization by circulating tumour cells. Nature.

[32] Zhang G, Miao F, Xu J, Wang R (2019). Mesenchymal stem cells from bone marrow regulate invasion and drug resistance of multiple myeloma cells by secreting chemokine CXCL13. Bosn. J. Basic Med. Sci..

[33] Atkinson SP (2012). A putative role for the immunoproteasome in the maintenance of pluripotency in human embryonic stem cells. Stem Cells.

[34] Hsieh JY, Fu YS, Chang SJ, Tsuang YH, Wang HW (2010). Functional module analysis reveals differential osteogenic and stemness potentials in human mesenchymal stem cells from bone marrow and Wharton's jelly of umbilical cord. Stem Cells Dev..

[35] Koike Y (2017). Anti-cell growth and anti-cancer stem cell activities of the non-canonical hedgehog inhibitor GANT61 in triple-negative breast cancer cells. Breast Cancer.

[36] Arnold KM, Flynn NJ, Sims-Mourtada J (2015). Activation of inflammatory responses correlate with hedgehog activation and precede expansion of cancer stem-like cells in an animal model of residual triple negative breast cancer after neoadjuvant chemotherapy. Cancer Stud. Mol. Med..

[37] Sansone P, Berishaj M, Rajasekhar VK, Ceccarelli C, Chang Q, Strillacci A (2017). Evolution of cancer stem-like cells in endocrine-resistant metastatic breast cancers is mediated by stromal microvesicles. Cancer Res..

[38] Sansone P (2016). Self-renewal of CD133(hi) cells by IL6/Notch3 signalling regulates endocrine resistance in metastatic breast cancer. Nat. Commun..

[39] Delbridge AR, Grabow S, Strasser A, Vaux DL (2016). Thirty years of BCL-2: translating cell death discoveries into novel cancer therapies. Nat. Rev. Cancer.

[40] Marotta LL (2011). The JAK2/STAT3 signaling pathway is required for growth of CD44(+)CD24(-) stem cell-like breast cancer cells in human tumors. J. Clin. Investig..

[41] Kim SY (2013). Role of the IL-6-JAK1-STAT3-Oct-4 pathway in the conversion of non-stem cancer cells into cancer stem-like cells. Cell Signal..

[42] Habib JG, O'Shaughnessy JA (2016). The hedgehog pathway in triple-negative breast cancer. Cancer Med..

[43] Krishnamurthy N, Kurzrock R (2018). Targeting the Wnt/beta-catenin pathway in cancer: update on effectors and inhibitors. Cancer Treat. Rev..

[44] Tamagnone L, Zacchigna S, Rehman M (2018). Taming the notch transcriptional regulator for cancer therapy. Molecules.

[45] Diboun I, Wernisch L, Orengo CA, Koltzenburg M (2006). Microarray analysis after RNA amplification can detect pronounced differences in gene expression using limma. BMC Genom..

[46] Tibshirani R (1997). The lasso method for variable selection in the Cox model. Stat. Med..

[47] Iasonos A, Schrag D, Raj GV, Panageas KS (2008). How to build and interpret a nomogram for cancer prognosis. J. Clin. Oncol..

[48] Heagerty PJ, Lumley T, Pepe MS (2000). Time-dependent ROC curves for censored survival data and a diagnostic marker. Biometrics.

[49] Subramanian A (2005). Gene set enrichment analysis: a knowledge-based approach for interpreting genome-wide expression profiles. Proc. Natl. Acad. Sci. USA.

