# Supplementary material for: Assessing the prognostic value of stemness-related genes in breast cancer patients
Source: Sci Rep. 2020 Oct 27;10:18325. doi: 10.1038/s41598-020-73164-3 (PMC7591576; doi:10.1038/s41598-020-73164-3)
Supplement: Supplementary file 1 — Supplementary Figures. [file 41598_2020_73164_MOESM1_ESM.pdf]

Assessing the prognostic value of stemness related genes in breast cancer patients

Wen-jie Wang, Han Wang, Mengsen Wang, Yue-qing Huang, Yu-Yuan Ma, Jie Qi, Jian-ping Shi, Wei Li

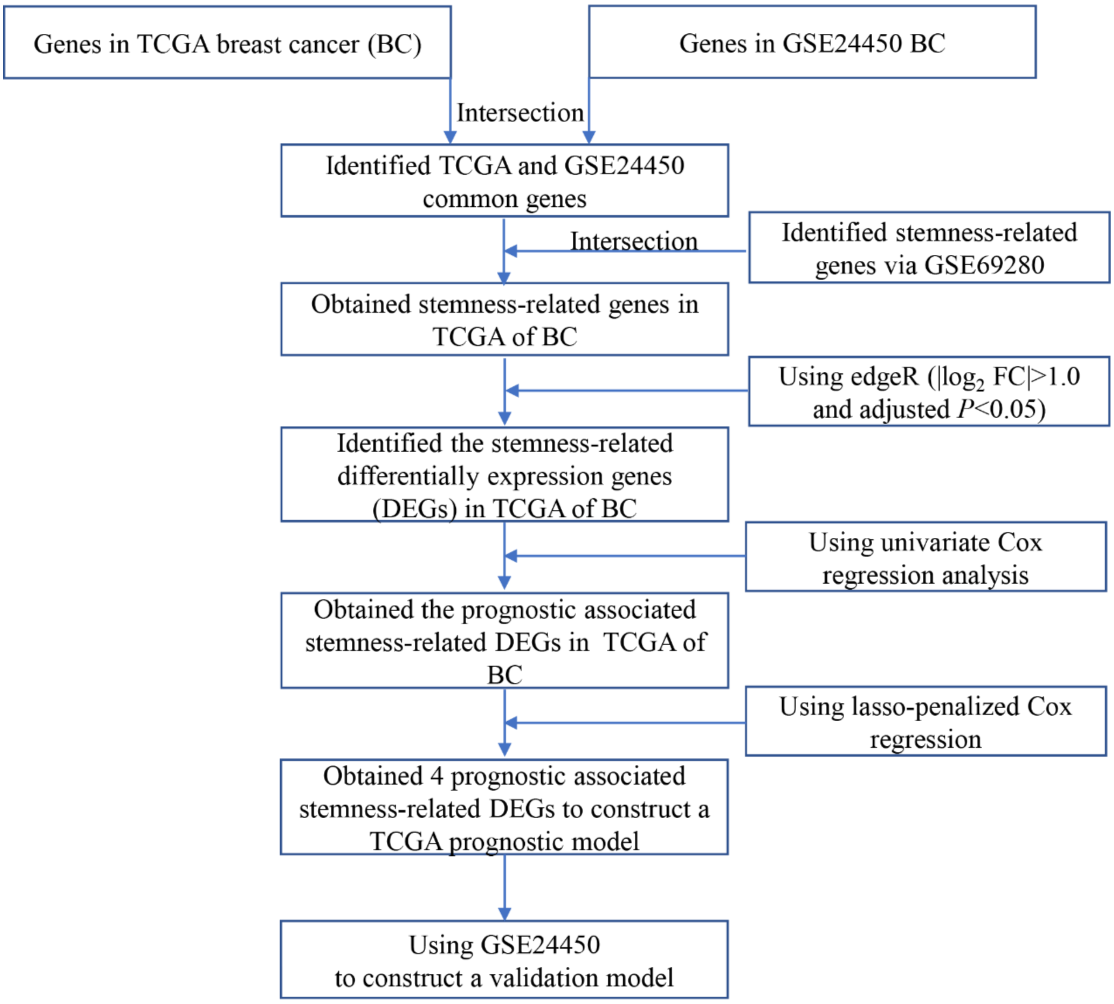

Fig S1. Flow chart

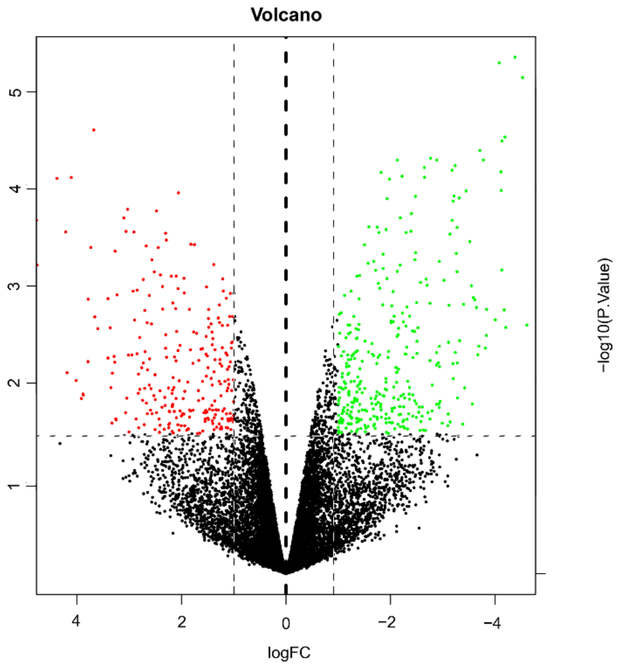

Fig S2. Identification of stemness-related differentially expressed genes in GSE69280.
